# Prepulse Inhibition of the Acoustic Startle Reflex in High Functioning Autism

**DOI:** 10.1371/journal.pone.0092372

**Published:** 2014-03-18

**Authors:** Sina Kohl, Carolin Wolters, Theo O. J. Gruendler, Kai Vogeley, Joachim Klosterkötter, Jens Kuhn

**Affiliations:** 1 Department for Psychiatry and Psychotherapy, University Hospital of Cologne, Cologne, North Rhine-Westphalia, Germany; 2 Department of Economics, Otto-von-Guericke-University, Magdeburg, Saxony-Anhalt, Germany; 3 Center for Behavioral Brain Sciences, Magdeburg, Saxony-Anhalt, Germany; 4 Institute for Neuroscience and Medicine – Cognitive Neuroscience (INM3), Research Center Juelich, Juelich, North Rhine-Westphalia, Germany; National Institutes of Health/NICHD, United States of America

## Abstract

**Background:**

High functioning autism is an autism spectrum disorder that is characterized by deficits in social interaction and communication as well as repetitive and restrictive behavior while intelligence and general cognitive functioning are preserved. According to the weak central coherence account, individuals with autism tend to process information detail-focused at the expense of global form. This processing bias might be reflected by deficits in sensorimotor gating, a mechanism that prevents overstimulation during the transformation of sensory input into motor action. Prepulse inhibition is an operational measure of sensorimotor gating, which indicates an extensive attenuation of the startle reflex that occurs when a startling pulse is preceded by a weaker stimulus, the prepulse.

**Methods:**

In the present study, prepulse inhibition of acoustic startle was compared between 17 adults with high functioning autism and 17 sex-, age-, and intelligence-matched controls by means of electromyography.

**Results:**

Results indicate that participants with high functioning autism exhibited significantly higher startle amplitudes than the control group. However, groups did not differ with regard to PPI or habituation of startle.

**Discussion:**

These findings challenge the results of two previous studies that reported prepulse inhibition deficits in high-functioning autism and suggest that sensorimotor gating is only impaired in certain subgroups with autism spectrum disorder.

## Introduction

In Leo Kanner's [1] original description of autism, he made the following observation:

“Another intrusion comes from loud noises and moving objects, which are therefore reacted to with horror. Tricycles, swings, elevators, vacuum cleaners, running water, gas burners, mechanical toys, egg beaters, even the wind could on occasions bring about a major panic. (…) Yet it is not the noise or motion itself that is dreaded. The disturbance comes from the noise or motion that intrudes itself, or threatens to intrude itself, upon the child's aloneness.” (p. 245)

In fact, about 90% of children with an autism spectrum disorder (ASD) exhibit sensory abnormalities [2], [3], [4]. Many of those show auditory hypersensitivity [5], [6], [7]. It was suggested that hypersensitivity is associated with an exceptional attention to detail in autism [5]. The weak central coherence theory of autism hypothesizes that the detail-focused processing style in children and adults with autism leads to a failure of central processing, which involves difficulties to extract global form or meaning [8]. Research regarding acoustic stimulation suggests that persons suffering from ASD are less susceptible to interference from melodic structure in music processing and to interference from visual to auditory perception (i.e. the *McGurk effect*). In addition, persons with autism show superior pitch and loudness processing and less distinctive auditory filtering [9], [10]. Behavioral experiments showed that persons with ASD require a higher signal-to-noise ratio in comparison to control subjects to perceive noise or speech [11], [12], [13]. In addition, individuals with ASD have difficulties ignoring distracting sounds in peripheral spatial locations [14] and segregating incoming sounds [15].

Abnormal sensory processing may lead to sensorimotor integration deficits [16], which have been shown across different sensory modalities in ASD [17]. Sensorimotor gating is an adaptive mechanism that prevents overstimulation within the transformation of sensory input into motor action [18]. Abnormalities of motor behavior are highly prevalent in autism and have a great impact on everyday life abilities [19]. However, the etiology of these symptoms remains unclear [20].

In the present study, prepulse inhibition (PPI) of the acoustic startle reflex is used as a proxy of sensorimotor gating. PPI indicates an extensive attenuation of the startle reflex that occurs when a startle eliciting stimulus, the pulse, is preceded by a weaker stimulus, the prepulse, within a timeframe of 30–500 ms [21]. More than 90% of healthy subjects show a reduction of 40–80% of the startle reflex, if a pulse is preceded by a prepulse that does not elicit a startle response itself [22]. The protection of processing hypothesis proposes that the processing of sensory stimuli is protected against interference through other irrelevant or distracting stimuli by preattentive mechanisms [21].

The present study aimed at exploring sensorimotor gating in high functioning autism (HFA), a subgroup of ASD that is characterized by social and communication deficits as well as repetitive, restrictive and stereotyped behavior, interests, and activities with preservation of intelligence and general cognitive functioning [23].

We could identify five studies that focused on PPI deficits in ASD patients. McAlonan and colleagues [24] as well as Perry and colleagues [25] found attenuated PPI in adults with high functioning autism and Asperger syndrome. In contrast, three other groups did not find any significant differences in PPI in samples that included children [26] or children as well as adolescents [27], [28]. Motivated by the inconsistent literature and with the idea that repetitive thoughts, behavior and activities might be a product of malfunctioning inhibition, it seemed important to us to conduct a study of high methodological quality in order to examine processing of auditory startle stimuli in a clearly defined subgroup of ASD patients. Derived from the above mentioned considerations we hypothesize that: participants with HFA will exhibit higher amplitudes of the startle reflex accompanied by reduced PPI in comparison to healthy controls.

## Materials and Methods

The study was approved by the local ethics committee of the Medical Faculty of the University of Cologne, and conducted in accordance with the declaration of Helsinki. The ethics committee was also involved in compiling the patient information and consent form. All subjects were informed in speech and writing about the purpose and procedure of the study and had to give their written informed consent in order to participate. Also approved by the local ethics committee, a very experienced psychiatrist and expert in the field of autism spectrum disorders (KV) gave the diagnosis and assessed the capacity of each participant to give his/her consent. As further support of the evaluation and for diagnostic purpose an intelligence test has been conducted with each participant (German multiple choice test *Wortschatztest* (WST)) to confirm ability to give consent. We did not obtain any surrogate consents.

### Materials

Autistic traits were estimated using the self-assessment questionnaire autism quotient (AQ) as screening instrument [29]. Empathy was measured by the self-report questionnaire empathy quotient (EQ) [30], and the tendency to systemize was acquired using the self-report questionnaire systemizing quotient (SQ) [31]. The Beck Depression Inventory (BDI) [32] was completed by all participants in order to measure depressive symptomatology. Intelligence was measured using the German multiple choice test *Wortschatztest (WST)* [33], which provides a short and valid estimation of general intelligence [34]. In addition, participants were asked to fill out a questionnaire, which included questions about any regular intake of medication, and the frequency and last point of time they consumed caffeine, alcohol, cannabis, and other drugs.

### Subjects

23 subjects with HFA and 23 normal control (NC) subjects (both groups: 7 women, 16 men) were assessed. The experimental group was diagnosed and recruited in the Autism Outpatient Clinic of the Department of Psychiatry, University of Cologne. The diagnosis was made by two experienced clinicians in two independent interviews (one of them KV) according to the International Classification of mental disorders [23] and supplemented by comprehensive neuropsychological assessment. Only individuals with the diagnosis of HFA (F84.5) were included into the sample. Formally, criteria of the DSM-IV [35] for HFA were met as well.

Control subjects were matched to sex, age, and intelligence scores of the experimental group. In our screening procedure, none of the control subjects had a BDI score above the clinically critical value of 17 [36] or an AQ above the cutoff score of 32 suggested by Baron-Cohen, Wheelwright, Skinner, Martin, and Clubley [37]. None of the control subjects showed any signs or symptoms of any possible psychiatric comorbid disorder. According to self disclosure, none of the participants suffered from any kind of hearing impairment. All of the subjects had an intelligence quotient (IQ) above 70 (see table 1).

**Table 1:** Participants' characteristics.

	HFA group		Control group		Statistical differences		
**Variable**	*M* (*SD*)	Range	*M* (*SD*)	Range	*T*	*df*	*p*
**Age**	40.2 (9.93)	23–53	43.4 (10.55)	26–56	.090	32	.373
**Education**	18.2 (5.48)	10–30	20.4 (3.35)	15–27	1.38	32	.178
**IQ**	116.4 (16.14)	89–143	119.2 (7.0)	101–133	.66	22	.515
**BDI**	11 (9.76)	0–34	5.1 (4.98)	0–15	2.24	24	.035*
**AQ**	41.1 (3.64)	34–45	15.9 (7.05)	5–29	13.09	24	<.001*
**EQ**	16.9 (7.82)	6–30	42.4 (12.83)	21–65	3.77	23	<.001*
**SQ**	37.3 (15.42)	11–67	21.7 (7.24)	11–36	7.01	32	<.001*

HFA =  High functioning autism; Education =  Years of education; IQ  =  Intelligence Quotient; BDI  =  Beck Depression Inventory; AQ  =  Autism Quotient; EQ  =  Empathy Quotient; SQ  =  Systemizing Quotient; *significant at an alpha level of .05.

Three of the subjects with HFA and three of the NC subjects had to be excluded from the data analysis because they did not exhibit sufficient startle responses and were thus classified as non-responders. One participant with HFA had to be excluded from further data analysis because he fell asleep during the test sessions, and one data set of an HFA subject was not usable due to technical malfunction. Additionally, one of the subjects with HFA and three of the NC subjects were excluded because visual inspection of the data indicated startle responses to the prepulses and PPI was therefore not reliably calculable.

Thus, *n* = 17 subjects with HFA and *n* = 17 NC subjects (each with 5 women, 12 men) remained in the sample (Table 1–3). There were no significant group differences regarding age, intelligence, and years of education. However, the HFA group showed significantly higher BDI scores than the control group. In addition, participants with HFA showed significantly higher AQ and SQ scores, and significantly lower EQ scores than NC participants.

**Table 2:** Comorbid psychiatric disorders.

Number of participants (group)	Psychiatric disorder
1 (HFA)	Bipolar disorder
1 (HFA)	Depression
1 (HFA)	Dysthymia
1 (HFA)	Anxiety Disorder
2 (HFA)	ADHD
1 (HFA)	ADD

HFA =  High functioning autism; ADHD  =  attention deficit/hyperactivity disorder; ADD  =  attention deficit disorder.

**Table 3:** Medication.

Number of participants	Medication
5 (HFA), 1 (NC)	Antidepressant drug (2× Sertraline, 2× Mirtazepine,
	1× Citalopram, 1× Moclobemid)
2 (HFA), 1 (NC)	Beta blocker (1× Bisoprolol, 1× Metahexal,
	1× Mesoprolol)
1 (HFA), 1 (NC)	Irbesartan
1 (NC)	Ramiprile
1 (HFA)	Lamotrigine

HFA =  High functioning autism; NC =  normal controls.

### Procedure

The startle reflex was measured in a quiet laboratory by means of an EMG of the musculus orbicularis oculi (EMG SR-HLAB, San Diego Instruments, San Diego, CA). To this end, two miniature electrodes were placed below and next to the participant's right eye, and a ground electrode was placed behind the right ear. Participants were briefed to sit quietly in an armchair and to fix their gaze on a spot. The stimulus material was presented binaurally through headphones (TDH-39-P, Maico, Minneapolis, MN). During the course of the measurement, background white noise of 70 dB was presented. Acoustic stimuli consisted of bursts of broadband white noise. Startle eliciting stimuli were presented for 40 ms at intensities of 110 dB with uncontrolled instant rise time, while prepulses were presented for 20 ms at 80 dB. Each PPI session started with a 5 min acclimation period of white noise. The first and the last block of the measurement comprised 5 pulse-alone trials. Intermittently, 10 pulse-alone trials, 10 prepulse-alone trials, and 30 prepulse-pulse trials were presented in a pseudorandom order. Prepulse-pulse stimulus onset asynchronies (SOAs) varied between 60, 120, and 240 ms. Intertrial intervals were 10, 15, 20, or 25 sec. Each trial was preceded by a baseline period of 60 ms, starting 80 ms before the stimulus onset.

### Data analyses

EMG data were analyzed using a *Matlab* program (MathWorks, Inc., Natick, MA) that was developed in our clinic in order to visually inspect and calculate PPI data. Recorded EMG activity was high-pass filtered at 30 Hz and low-pass filtered at 300 Hz using a 4^th^ order butterworth filter, and a 50 Hz notch filter with a width of 6 Hz was used to reduce power line interference (hier bitte den van Boxtel Artikel zitieren). The EMG signal was then rectified and smoothed using a 10 point moving average. By means of visual inspection, any trial featuring excessive noise in the EMG signal or a spontaneous blink in the period immediately preceding the stimulus onset or the minimal response onset were excluded from further analyses. Based on these criteria, 9.44% of trials had to be excluded from data analysis. In addition, the first two pulse-alone trials of each PPI measure were excluded from the analysis, as it was assumed that these trials evoke unrepresentatively high startle amplitudes [38].

Criteria for qualifying the EMG signal as an actual startle response were defined in accordance with guidelines for human startle eye blink EMG studies [38]. The latency window was set at 20 to 150 ms after pulse onset, and the minimum response amplitude was set at 2 *SD* above baseline. The response peak was identified by a computerized algorithm. If visual inspection indicated that response onset was outside the latency window or the criteria for minimal response size was not met, the trial was classified as a zero response trial. These trials were not considered in the calculation of the specific values. Participants were identified as non-responders and therefore excluded if they exhibited startle responses in less than half of the pulse-alone trials. Participants for whom visual inspection of the prepulse-alone trials revealed distinct reactions to the prepulses in half or more of the trials were excluded as well.

### Statistical analyses

A commercial software package was used for statistical analysis of the data (SPSS for windows, version 21, IBM Corp., Armonk, NY).

#### Startle amplitudes

Mean startle amplitudes were calculated by averaging the response peaks of all included trials, and compared between groups using an unpaired Student's *t*-test. Differences in startle amplitudes within the different trial conditions were compared by means of a one-way repeated-measure ANOVA. In addition to startle amplitudes we calculated the response probability, the probability that a valid startle response follows the startle eliciting pulse. The response probability is given in percent.

#### PPI

Percentage PPI values were calculated according to the following formula: 100 – [(mean amplitude prepulse trials/mean amplitude pulse alone trials) x 100]. Besides comparing PPI between the HFA and NC group, PPI group differences were assessed using three different one-way repeated measures ANOVAs, including the between-subjects factor of group (1. male/female; 2. smoker/non-smoker; 3. HFA/NC), and within-subjects repeated measures of PPI across the different trial types. Mean startle amplitude across all pulse-alone trials was included as a covariate. Furthermore, PPI differences across all three types of trials were calculated using Student's *t*-tests.

#### Correlations between PPI and autistic traits

To assess the relationship between PPI and autistic traits, the AQ, EQ, and SQ scores of the HFA subjects were correlated with percentage PPI of the three trial types using the Pearson's correlation coefficient.

#### Habituation

Primarily, group differences in habituation of the startle response across all pulse-alone trials were calculated. To this end, the altogether 20 pulse-alone trials were split into four blocks of five trials each. A one-way repeated measures ANOVA was conducted, including the between-subjects factor of diagnosis (HFA/NC) and the within-subjects factor of mean startle amplitude of the four pulse-alone blocks. Additionally, the overall habituation rate was examined by calculating the percent decrease from mean startle amplitude in the first five pulse-alone trials to mean amplitude in the last five trials and compared between groups by means of an unpaired Student's *t*-test.

Before conducting the ANOVAs, the assumption of sphericity was tested for the data, and degrees of freedom were corrected using Greenhouse-Geisser estimates of sphericity, if necessary. Effect sizes for ANOVAs were calculated using η^2^_partial_, effect sizes for post-hoc pairwise comparisons were calculated using Cohen's *d*.

## Results

### Startle amplitudes

Analysis of startle amplitudes for pulse-alone trials revealed significantly higher startle amplitudes (*M* = 179.1, *SD* = 146.93) for HFA patients than control subjects (*M* = 96.6, *SD* = 62.93), *T*(32) = 2.13, *p* = .041. The mean response probability for a significant startle amplitude in pulse alone trials for the HFA group *(M* = 93.66 *SD = *9.04) was significantly higher compared to the control group (*M* = 80.52, *SD* = 18.81), *T*(32) = 2.6, *p* = .014.

### PPI

Examining PPI differences between the HFA and NC group, Mauchly's test of sphericity was not significant, χ^2^ = .17, *df* = 2, *p* = .435. There was a significant main effect of trial type (60/120/240 ms SOA), *F*(2) = 3.35, *p* = .041, η^2^_partial_ = .098. Post-hoc pairwise comparisons indicated that PPI was significantly lower in trials with an SOA of 240 ms than in those with an SOA of 60 ms, *p* = .015, *d* = .61, and 120 ms, *p* = .001, *d* = .63 (*p*-values were Bonferroni adjusted).

**Figure 1:**
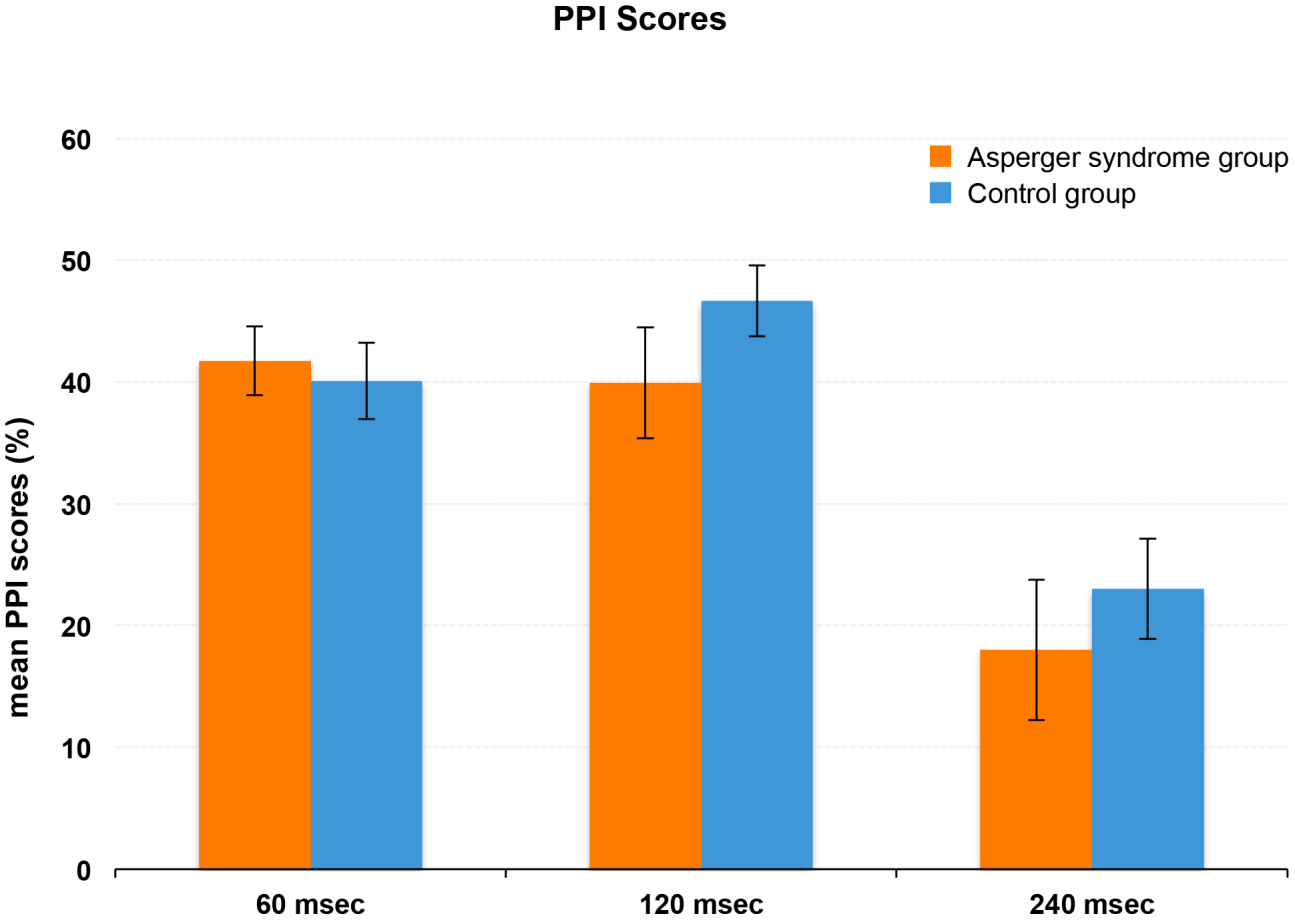
PPI scores. Mean PPI scores (± SEM) across the three trial conditions in subjects with high functioning autism (HFA) and normal control (NC) subjects.

However, there was no significant effect of mean startle amplitude across pulse-alone trials, *F*(1) = .30, *p* = .590, and no significant interaction of startle amplitude and trial type, *F*(2) = .12, *p* = .887. Equally, there was no significant main effect of diagnosis on PPI, *F*(1) = .02, *p* = .879, and no significant interaction of diagnosis and trial type, *F*(2) = .13, *p* = .877. Figure 1 shows the PPI values of HFA and NC subjects across the three different types of trials. Likewise, there were no differences in overall PPI between participants with HFA (*M* = 32.5, *SD* = 31.11) and control participants (M = 36.8, SD = 22.11), T(32) = .46, p = .649.

The response probabilities for the prepulse trials did not differ significantly between the HFA Group (*M* = 84.18, *SD* = 18.73) and the controls (*M* = 72.66, *SD* = 24.18), *T*(32) = 1.55, *p* = .13.

### Correlations between autistic traits and PPI

There were no significant correlations between AQ, EQ, and SQ scores and PPI in the different trial types among HFA participants (all *p*>.1).

### Habituation

**Figure 2:**
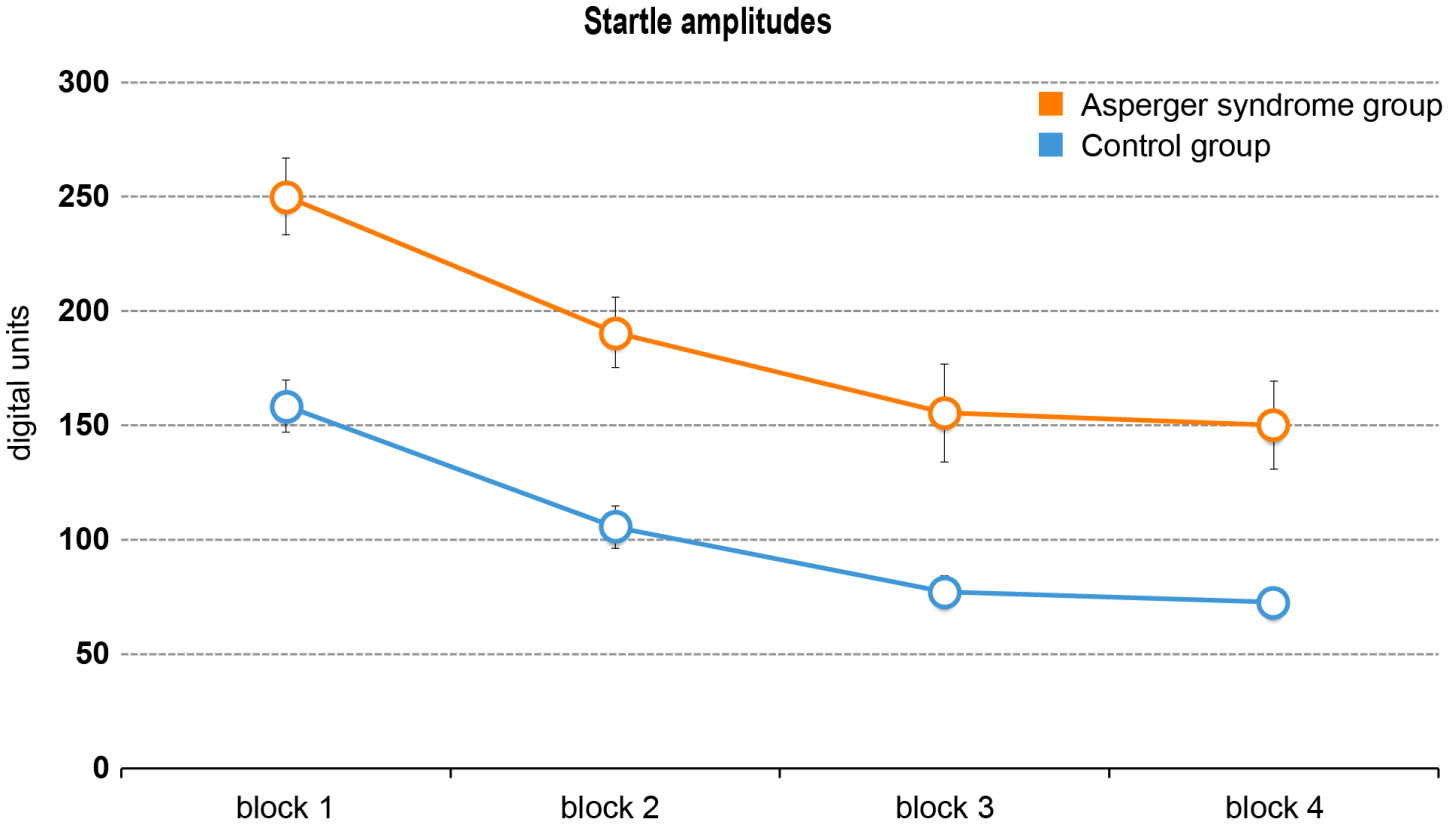
Startle amplitudes and habituation rates. Habituation rates across the four blocks of trials in the high functioning autism (HFA) and normal control (NC) group. Mean startle amplitudes (± SEM) of each five pulse-alone trials are shown. HFA participants showed significantly higher absolute startle amplitudes compared to NC participants, while habituation rates were similar.

Mauchly's test indicated that the assumption of sphericity was not met for startle amplitude scores, χ^2^ = 23.52, *df* = 5, *p*<.001. Therefore, degrees of freedom were corrected using Greenhouse-Geisser estimates of sphericity. Results showed that the startle amplitude was significantly affected by block, *F*(2) = 30.32, *p*<.001, η^2^_partial_ = .486, indicating habituation across the four blocks of pulse-alone trials. Pairwise comparisons determined that the mean startle amplitude decreased from the first block to the second, *p*<.001, *d* = .47, to the third, *p*<.001, *d* = .67, and to the fourth block, *p*<.001, *d* = .75. In addition, the startle amplitude significantly decreased from the second to the fourth block, *p*<.001, *d* = .31 (*p*-values were Bonferroni adjusted). Data analysis also revealed a significant main effect of diagnosis, *F*(1) = 4.67, *p = *.038, η^2^_partial_ = .127, reflecting the differences in startle amplitude between HFA and NC participants. However, there was no significant block-by-group effect, *F*(2) = .19, *p* = .834. Visual inspection confirmed that the two groups had similar decreases in startle amplitude over the four blocks (see figure 2).

Analysis of overall percentage habituation from block 1 to block 4 did not show significant differences between HFA (*M* = 48.0, *SD* = 26.31) and NC participants (*M* = 52.1, *SD* = 25.13), *T*(32) = .47, *p* = .641.

## Discussion

The present study assessed auditory sensorimotor gating by means of the acoustic startle reflex in subjects with HFA. Our results indicate significantly higher mean startle amplitudes in the HFA group and for the pulse alone condition significantly higher response probabilities compared to the control group. But groups did not differ significantly in terms of PPI, neither in the different SOA trial types, nor across all trials.

### Startle amplitude

Increased startle amplitudes among HFA subjects may be caused by auditory hypersensitivity, which often occurs in autism [5]. Abnormalities in the processing of auditory information were also shown by neurophysiologic studies finding atypical neural activity in early auditory pathways in ASD [39]. Magnetoencephalography studies have linked auditory hypersensitivity to maturational abnormalities in the auditory cortex in autism, resulting in delayed responses of the auditory cortex to acoustic stimuli [40], and abnormalities in myelination processes, causing slower transmission rates in central auditory pathways [41], [42]. In addition, reduced interneuron activity might lead to abnormal brain connectivity, which might in turn cause cortical hypersensitivity in the primary auditory cortex [43].

### PPI

The other major hypothesis could not be proven by our results. Bearing in mind the weak central coherence theory of autism we hypothesized, subjects with HFA would show PPI deficits in comparison to the control group. But in accordance with three other studies [26], [27], [28] our results indicate that PPI does not differ between the HFA and the NC group. However, the aforementioned studies examined PPI in samples of children and young adults of different age on the autistic spectrum. As PPI is thought to mature with advancing age in childhood [44], PPI deficits might vary between children of different age groups, or may not be observable in children at all.

So far, only one study explicitly focused on adults with HFA [24]. The authors found significant PPI differences between subjects with HFA and the control group in only one condition (SOA 120 ms), while PPI in the HFA group was similar to or even higher than that of the control group in the other conditions (SOA 30 and 60 ms). In contrast, Perry et al. [25] found significantly reduced PPI in subjects with high-functioning autism in comparison to control subjects in the SOA 60 ms condition, but not in the SOA 30 and 120 ms conditions. However, these authors did not report the specific diagnoses of their participants. It must be emphasized that our cohort differs from the autism groups of previous studies in terms of age, sex, and IQ. These differences might have implications for our results. For example, our cohort has a higher mean IQ compared to previous studies, this might imply that our cohort is composed of pure HFA and not confounded by low intelligence or other cognitive dysfunctions.

### Habituation

Even though individuals with HFA exhibited higher startle amplitudes than control subjects, habituation rates were similar in both groups. This finding is consistent with those of Ornitz et al. [27] and [24], who did not find any differences in habituation between subjects with ASD and control subjects. In contrast, Perry et al. [25] found that subjects with ASD show decelerated habituation, speculating that reduced habituation might lead to behavioral and cognitive deficits in autism. However, their sample comprised only male subjects with ASD, while the current study examined men and women with HFA. Then again, previous studies found reduced habituation to acoustic stimuli in children with autism [45] and at high risk for autism [46]. A cross-sectional questionnaire study found that less developmentally mature children with autism and other developmental disorders exhibited the highest rates of sensory processing difficulties [47]. Perhaps, habituation to sensory stimuli is associated with mental age, explaining normal habituation rates in normally intelligent adults with HFA.

### Study limitations

Some factors might limit the degree to which the findings of the present study can be generalized. The determination of unimpaired hearing, potential psychiatric and neurological disorders as well as use of medication relied on self-report only. Therefore, information might be incomplete or unreliable. Seven HFA subjects reported an additional mental disorder. Studies consistently reported no alterations of PPI in depression and ADHD [48]. In bipolar disorder, PPI was found to be state dependent, while evidence regarding PPI deficits in anxiety disorders is still poor [48]. Although unlikely it can thus not completely be ruled out that comorbid disorders had an effect on sensorimotor gating. Drug intake of some participants in both groups might have influenced PPI, but here too an influence is more unlikely as animal and human studies showed that antidepressant drugs do not alter PPI [49]. However, evidence regarding the effects of other drugs, such as beta blockers, on PPI is still poor. In order to rule out such effects, subjects with comorbid psychiatric or neurological disorders and with drug intake should be excluded for less ambiguous results. Further, the mean PPI value of the healthy control group was relatively low compared to previous studies, which report PPI values between 40 to 80% in 90% of healthy adults [22]. Therefore there is a chance that comparison of control group and HFA group did not significantly differ due to relatively low PPI in controls.

### Conclusions

While the results indicated no sensorimotor gating deficits, analysis of startle amplitudes revealed abnormalities in terms of exaggerated startle reaction in subjects with HFA. Individuals with HFA showed higher startle amplitudes than NC subjects, but exhibited similar rates of PPI and habituation of startle. These findings argue for functional pathophysiology on the level of the primary startle pathway, involving structures such as the caudal pontine reticular nucleus and ventrolateral tegmental nucleus in HFA. In contrast, the PPI circuit, including the pedunculopontine tegmental nucleus and inferior and superior colliculi, seems to be unimpaired.

Therefore, the extreme responses in individuals with autism to sensory stimulation that were already observed by Kanner in 1943 [1] are probably mainly due to a general hypersensitivity, but not difficulties in sensorimotor gating. Individuals with autism thus show unimpaired habituation of sensory stimuli and unimpaired transformation of these stimuli into motor action, while generally exhibiting more pronounced motor responses to sensory input in comparison to the healthy population.

The present study is the first to demonstrate normal PPI and habituation in a sample of adults with high-functioning autism. In consideration of previous studies, this finding suggests that sensorimotor gating deficits are not a distinctive feature of autism, but rather occur only in certain subgroups. For example, Yuhas et al. [28] found attenuated PPI in subjects with autism and Fragile-X-syndrome (FXS) in comparison to subjects with idiopathic autism and control subjects, indicating that FXS might be responsible for sensorimotor deficits in some individuals with autism. Therefore, future studies should focus on differential sample characteristics that might influence PPI, such as the occurrence of FXS. In order to minimize the possibility of type II errors, large sample sizes are needed. Future studies should particularly pay attention to careful sample selection and methodological aspects, as differences in methodology between the existing studies, such as reporting magnitude versus amplitude of the startle response, or different mean IQ or gender distributions, make a definite evaluation at this point in time impossible.

Moreover, it would be interesting to further explore the association between sensory processing and sensorimotor gating in autism. One possibility to explore sensory processing along with PPI is to measure gating of the auditory evoked potential by means of EEG [26]. Even though these authors did not find any significant differences between children and adolescents with and without autism, future research should examine possible associations between sensory gating and sensorimotor gating in higher-functioning adults.

## References

[1] Kanner L (1943) Autistic disturbances in affective contact. Nervous child 2: 217–250.

[2] Kern JK, Trivedi MH, Garver CR, Grannemann BD, Andrews AA, et al (2006) The pattern of sensory processing abnormalities in autism. Autism 10: 480–494. 16940314 10.1177/1362361306066564

[3] Leekam SR, Neito C, Libby SJ, Wing L, Gould J (2001) Describing the sensory abnormalities of children and adults with autism. Journal of Autism Developmental Disorders 37: 894–910. 10.1007/s10803-006-0218-7 17016677

[4] Tomchek SD, Dunn W (2007) Sensory processing in children with and without autism: a comparative study using the short sensory profile. Am J Occup Ther 61: 190–200. 17436841 10.5014/ajot.61.2.190

[5] Baron-Cohen S, Ashwin E, Ashwin C, Tavassoli T, Chakrabarti B (2009) Talent in autism: hyper-systemizing, hyper-attention to detail and sensory hypersensitivity. Philos Trans R Soc Lond B Biol Sci 364: 1377–1383. 19528020 10.1098/rstb.2008.0337 PMC2677592

[6] Gomes E, Pedroso FS, Wagner MB (2008) Auditory hypersensitivity in the autistic spectrum disorder. Pro Fono 20: 279–284. 19142473 10.1590/s0104-56872008000400013

[7] Hitoglou M, Ververi A, Antoniadis A, Zafeiriou DI (2010) Childhood autism and auditory system abnormalities. Pediatr Neurol 42: 309–314. 20399382 10.1016/j.pediatrneurol.2009.10.009

[8] Frith U (1989) Autism: explaining the Enigma: Blackwell.

[9] O'Connor K (2012) Auditory processing in autism spectrum disorder: a review. Neurosci Biobehav Rev 36: 836–854. 22155284 10.1016/j.neubiorev.2011.11.008

[10] Ouimet T, Foster NE, Tryfon A, Hyde KL (2012) Auditory-musical processing in autism spectrum disorders: a review of behavioral and brain imaging studies. Ann N Y Acad Sci 1252: 325–331. 22524375 10.1111/j.1749-6632.2012.06453.x

[11] Alcantara JI, Weisblatt EJ, Moore BC, Bolton PF (2004) Speech-in-noise perception in high-functioning individuals with autism or Asperger's syndrome. J Child Psychol Psychiatry 45: 1107–1114. 15257667 10.1111/j.1469-7610.2004.t01-1-00303.x

[12] DePape AM, Hall GB, Tillmann B, Trainor LJ (2012) Auditory processing in high-functioning adolescents with Autism Spectrum Disorder. PLoS One 7: e44084. 22984462 10.1371/journal.pone.0044084 PMC3440400

[13] Groen WB, van Orsouw L, Huurne N, Swinkels S, van der Gaag RJ, et al (2009) Intact spectral but abnormal temporal processing of auditory stimuli in autism. J Autism Dev Disord 39: 742–750. 19148738 10.1007/s10803-008-0682-3

[14] Teder-Salejarvi WA, Pierce KL, Courchesne E, Hillyard SA (2005) Auditory spatial localization and attention deficits in autistic adults. Brain Res Cogn Brain Res 23: 221–234. 15820630 10.1016/j.cogbrainres.2004.10.021

[15] Lepisto T, Kuitunen A, Sussman E, Saalasti S, Jansson-Verkasalo E, et al (2009) Auditory stream segregation in children with Asperger syndrome. Biol Psychol 82: 301–307. 19751798 10.1016/j.biopsycho.2009.09.004 PMC2771139

[16] Takarae Y, Luna B, Minshew NJ, Sweeney JA (2008) Patterns of visual sensory and sensorimotor abnormalities in autism vary in relation to history of early language delay. J Int Neuropsychol Soc 14: 980–989. 18954478 10.1017/S1355617708081277 PMC2928719

[17] Fournier KA, Hass CJ, Naik SK, Lodha N, Cauraugh JH (2010) Motor coordination in autism spectrum disorders: a synthesis and meta-analysis. J Autism Dev Disord 40: 1227–1240. 20195737 10.1007/s10803-010-0981-3

[18] Swerdlow NR, Braff DL, Geyer MA (2000) Animal models of deficient sensorimotor gating: what we know, what we think we know, and what we hope to know soon. Behav Pharmacol 11: 185–204. 11103873 10.1097/00008877-200006000-00002

[19] Gowen E, Hamilton A (2013) Motor abilities in autism: a review using a computational context. J Autism Dev Disord 43: 323–344. 22723127 10.1007/s10803-012-1574-0

[20] Miyahara M (2013) Meta review of systematic and meta analytic reviews on movement differences, effect of movement based interventions, and the underlying neural mechanisms in autism spectrum disorder. Front Integr Neurosci 7: 16. 23532374 10.3389/fnint.2013.00016 PMC3607787

[21] Graham FK (1975) The More or Less Startling Effects of Weak Prestimulation. Psychophysiology 12: 238–248. 1153628 10.1111/j.1469-8986.1975.tb01284.x

[22] Filion DL, Dawson ME, Schell AM (1998) The psychological significance of human startle eyeblink modification: a review. Biol Psychol 47: 1–43. 9505132 10.1016/s0301-0511(97)00020-3

[23] Organization WH (1992) ICD-10 Classification of mental and behavioral disorders: Clinical descriptions and diagnostic guidelines. Geneva: World Helath Organization.

[24] McAlonan GM, Daly E, Kumari V, Critchley HD, van Amelsvoort T, et al (2002) Brain anatomy and sensorimotor gating in Asperger's syndrome. Brain 125: 1594–1606. 12077008 10.1093/brain/awf150

[25] Perry W, Minassian A, Lopez B, Maron L, Lincoln A (2007) Sensorimotor gating deficits in adults with autism. Biol Psychiatry 61: 482–486. 16460695 10.1016/j.biopsych.2005.09.025

[26] Oranje B, Lahuis B, van Engeland H, Jan van der Gaag R, Kemner C (2013) Sensory and sensorimotor gating in children with multiple complex developmental disorders (MCDD) and autism. Psychiatry Res 206: 287–292. 23164481 10.1016/j.psychres.2012.10.014

[27] Ornitz EM, Lane SJ, Sugiyama T, de Traversay J (1993) Startle modulation studies in autism. J Autism Dev Disord 23: 619–637. 8106303 10.1007/BF01046105

[28] Yuhas J, Cordeiro L, Tassone F, Ballinger E, Schneider A, et al (2011) Brief report: Sensorimotor gating in idiopathic autism and autism associated with fragile X syndrome. J Autism Dev Disord 41: 248–253. 20521090 10.1007/s10803-010-1040-9 PMC3023021

[29] Baron-Cohen S, Hoekstra RA, Knickmeyer R, Wheelwright S (2006) The Autism-Spectrum Quotient (AQ)—adolescent version. J Autism Dev Disord 36: 343–350. 16552625 10.1007/s10803-006-0073-6

[30] Baron-Cohen S, Wheelwright S (2004) The empathy quotient: an investigation of adults with Asperger syndrome or high functioning autism, and normal sex differences. J Autism Dev Disord 34: 163–175. 15162935 10.1023/b:jadd.0000022607.19833.00

[31] Baron-Cohen S, Richler J, Bisarya D, Gurunathan N, Wheelwright S (2003) The systemizing quotient: an investigation of adults with Asperger syndrome or high-functioning autism, and normal sex differences. Philos Trans R Soc Lond B Biol Sci 358: 361–374. 12639333 10.1098/rstb.2002.1206 PMC1693117

[32] Hautzinger M, Bailer M, Worall H, Keller F (1995) Beck Depressions-Inventory (BDI). Bern: Huber.

[33] Schmidt K-H, Metzler P (1992) Wortschatztest [Vocabulary test (WST)]. Weinheim: Beltz Test GmbH.

[34] Lehrl S, Triebig G, Fischer B (1995) Multiple choice vocabulary test MWT as a valid and short test to estimate premorbid intelligence. Acta Neurol Scand 91: 335–345. 7639062 10.1111/j.1600-0404.1995.tb07018.x

[35] APA (2000) Diagnostic and Statistical Manual of Mental Disorders, foruth edition revised (DSM IV-TR). Washington, DC: American Psychiatric Association.

[36] Beck AT, Ward C, Mendelson M (1961) Beck Depression Inventory (BDI). Archives of General Psychiatry 4: 561–571. 13688369 10.1001/archpsyc.1961.01710120031004

[37] Baron-Cohen S, Wheelwright S, Skinner R, Martin J, Clubley E (2001) The autism-spectrum quotient (AQ): evidence from Asperger syndrome/high-functioning autism, males and females, scientists and mathematicians. J Autism Dev Disord 31: 5–17. 11439754 10.1023/a:1005653411471

[38] Blumenthal TD, Cuthbert BN, Filion DL, Hackley S, Lipp OV, et al (2005) Committee report: Guidelines for human startle eyeblink electromyographic studies. Psychophysiology 42: 1–15. 15720576 10.1111/j.1469-8986.2005.00271.x

[39] Marco EJ, Hinkley LB, Hill SS, Nagarajan SS (2011) Sensory processing in autism: a review of neurophysiologic findings. Pediatr Res 69: 48R–54R. 10.1203/PDR.0b013e3182130c54 PMC3086654 21289533

[40] Roberts TP, Khan SY, Rey M, Monroe JF, Cannon K, et al (2010) MEG detection of delayed auditory evoked responses in autism spectrum disorders: towards an imaging biomarker for autism. Autism Res 3: 8–18. 20063319 10.1002/aur.111 PMC3099241

[41] Bruneau N, Roux S, Adrien JL, Barthelemy C (1999) Auditory associative cortex dysfunction in children with autism: evidence from late auditory evoked potentials (N1 wave-T complex). Clin Neurophysiol 110: 1927–1934. 10576489 10.1016/s1388-2457(99)00149-2

[42] Gage NM, Siegel B, Roberts TP (2003) Cortical auditory system maturational abnormalities in children with autism disorder: an MEG investigation. Brain Res Dev Brain Res 144: 201–209. 12935917 10.1016/s0165-3806(03)00172-x

[43] Matsuzaki J, Kagitani-Shimono K, Goto T, Sanefuji W, Yamamoto T, et al (2012) Differential responses of primary auditory cortex in autistic spectrum disorder with auditory hypersensitivity. Neuroreport 23: 113–118. 22146579 10.1097/WNR.0b013e32834ebf44

[44] Gebhardt J, Schulz-Juergensen S, Eggert P (2012) Maturation of prepulse inhibition (PPI) in childhood. Psychophysiology 49: 484–488. 22176532 10.1111/j.1469-8986.2011.01323.x

[45] Kemner C, Orange B, Verbaten MN, van Engeland H (2002) Normal P50 gating in children with autism. Journal of Clinical Psychiatry 63: 214–217. 11926720 10.4088/jcp.v63n0307

[46] Guiraud JA, Kushnerenko E, Tomalski P, Davies K, Ribeiro H, et al (2011) Differential habituation to repeated sounds in infants at high risk for autism. Neuroreport 22: 845–849. 21934535 10.1097/WNR.0b013e32834c0bec

[47] Baranek GT, David FJ, Poe MD, Stone WL, Watson LR (2006) Sensory Experiences Questionnaire: discriminating sensory features in young children with autism, developmental delays, and typical development. J Child Psychol Psychiatry 47: 591–601. 16712636 10.1111/j.1469-7610.2005.01546.x

[48] Kohl S, Heekeren K, Klosterkotter J, Kuhn J (2013) Prepulse inhibition in psychiatric disorders—apart from schizophrenia. J Psychiatr Res 47: 445–452. 23287742 10.1016/j.jpsychires.2012.11.018

[49] Braff DL, Geyer MA, Swerdlow NR (2001) Human studies of prepulse inhibition of startle: normal subjects, patient groups, and pharmacological studies. Psychopharmacology (Berl) 156: 234–258. 11549226 10.1007/s002130100810

